# Horizontal Polarized DC Grounded Omnidirectional Antenna for UAV Ground Control Station

**DOI:** 10.3390/s21082763

**Published:** 2021-04-14

**Authors:** Muhammad Shahzad Sadiq, Cunjun Ruan, Hamza Nawaz, Shahid Ullah, Wenlong He

**Affiliations:** 1 School of Electronic and Information Engineering, Beihang University, Beijing 100191, China; shahzadsadiq@buaa.edu.cn (M.S.S.); shahidkhan@buaa.edu.cn (S.U.); 2 Beijing Key Laboratory for Microwave Sensing and Security Applications, Beihang University, Beijing 100191, China; 3 School of Electrical, Information and Electronics Engineering, Shanghai Jiao Tong University, Shanghai 200240, China; hamza_nawaz@hotmail.com; 4 College of Electronics and Information Engineering, Shenzhen University, Shenzhen 518060, China; wenlong.he@szu.edu.cn

**Keywords:** horizontal polarization, UAV ground station, Omni-directional

## Abstract

A new slot-based antenna design capable of producing horizontal polarization for unmanned aerial vehicle (UAV) ground control station (GCS) applications is outlined in this paper. The proposed antenna consists of oversize coaxial cylinders, slots, and slot-feed assembly. Each of the four vertical slots, arranged periodically around the antenna’s outer cylinder, emits a horizontally polarized broad beam of radiation, in phase, to produce an omnidirectional pattern. The antenna possesses a low-ripple ±0.5 dB in azimuth gain (yaw) due to its symmetric axis shape and an enclosed feed within itself, which does not radiate and interfere with the main azimuth pattern. This is crucial for a UAV GCS to symmetrically extend its coverage range in all directions against yaw planes. Simulation and measurement results reveal that the antenna maintains stable gain in the omnidirectional pattern (+0.5 dB) over the entire operational frequency band (2.55 GHz to 2.80 GHz), where S11 is lower than −10 dB. A further advantage of this configuration is its enhanced polarization purity of −40 dB over the full frequency band. The direct-current (DC) grounding approach used in this antenna is beneficial due to its electrostatic discharge (ESD) and lightning protection. Furthermore, its aerodynamic, self-supporting, and surface-mount structural shape makes this antenna a good and worthy choice for a UAV GCS.

## 1. Introduction

Since the discovery of the interdependency between electrical parameters and electromagnetic radiation, antennas have been developed that actively exploit this phenomenon. Antennas convert electrical parameters (current and voltages) into electromagnetic parameters (electric and magnetic fields) and vice versa. Hence, an antenna can be regarded as a transducer or a sensor as it converts electrical energy to electromagnetic energy, or the opposite [1]. Antennas are always considered essential parts of communication systems, and their radiation and polarization characteristics play a vital role in defining such systems’ performance and efficiency [2,3].

The use of unmanned aerial vehicles (UAVs) is rapidly expanding to commercial, scientific, agricultural, and military applications [4]. To overcome the difficulty of finding the exact location of mobile UAVs from ground control stations (GCS), omnidirectional antennas are utilized to resolve acquisition and pointing complications [5,6,7,8,9,10]. It has been proven that using horizontally polarized antennas can achieve a 10 dB improvement in terms of system gain as compared to vertically polarized antennas [11]. For GCS deployment, where the antenna is intended to cover a wide range of angles at variant distances, it is essential to utilize a low-gain ripple radiation pattern to ensure continuous coverage in the yaw plane (azimuth plane) [12,13,14] as gain ripple fluctuates and reduces the coverage range at variant horizontal plane angles [15].

The challenging aspect of designing horizontally polarized omnidirectional antennas is producing a uniform and in-phase current in the antenna’s azimuth plane. That necessary condition can be fulfilled by utilizing a single loop or multi-element arrangements [16]. Three primary topological schemes are used with omnidirectional horizontally polarized antennas. In the first topology, a single radiating structure, such as a loop, is utilized to achieve horizontally polarized radiations [17,18,19,20], but it is inherently band-limited due to an open feed. The second group imitates the loop arrangement of first with dipole elements arranged in a ring or circular array format [21,22,23,24,25,26] at the expense of a complex open feeding arrangement. The third topology utilizes slots to complete the horizontally polarized antenna. There are a few slot-based omnidirectional antennas described in the literature. In [27], an omnidirectional antenna operating at X band used an array of slot doublets etched in the broadside wall of the rectangular waveguide. However, there was no mention of the azimuth gain, gain fluctuations, and operating band. A slot-based antenna capable of producing horizontal polarization was constructed by arranging alternate slots with opposite tilt angles along the axis with intervals of λg/2. To improve the antenna’s performance, alternate axial slot arrays were shifted by λg/4 along the axis. Even then, it was not improved by more than −7 dB [28]. In [29], an omnidirectional antenna was proposed, but it was circularly polarized. Moreover, it was not direct-current (DC) grounded and had a built-in main beam frequency scanning problem. In [30], a slant polarized omnidirectional antenna was presented. All slot-based horizontal polarized topologies were arranged in a series of fed axial arrays to achieve the required polarization. The other two methodologies had open feeding networks that interfered with the radiating apertures and perturbed antenna radial symmetry causing an uneven azimuth gain pattern, which further reduced antenna coverage range.

This paper proposes an omnidirectional antenna capable of achieving low azimuthal gain variations of ±0.5 dB. This work is the first single-element design based on slots capable of horizontal polarization and stable gain without making a complex axial array to achieve the required polarization. The flaunted antenna comprises four slot apertures evenly spaced around the antenna’s outer circumference. It also encloses the feeding topology, so antenna symmetry is not disturbed. The device’s compactness, ruggedness, and direct-current grounding are further important features of this antenna design. The proposed technique has improved polarization stability since the cross-pols are very weak relative to the co-pols. The antenna structure is exhibited and explained in Section 2. Section 3 elaborates on the slot-feed mechanism. In Section 4, simulation verification is performed. Section 6 describes the manufacturing and measurements of the antenna prototype. Section 6 presents a comparison of the proposed work with those published. Finally, Section 7 details our conclusions.

## 2. Antenna

**Figure 1:**
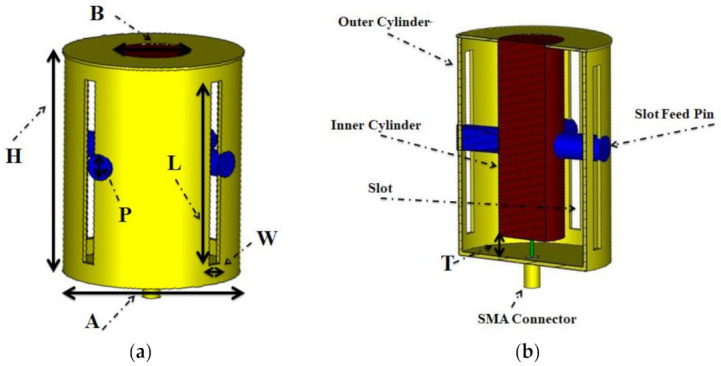
The geometry of the horizontally polarized omnidirectional antenna: (**a**) 3D view, (**b**) cross-sectional view.

The structure of the suggested horizontally polarized omnidirectional antenna is shown in Figure 1. It must be shaped like a pole due to vehicle-mounting requirements. It is based on the coaxial line and is composed of inner and outer conductors. There are etched slots around the coaxial cylinder, and the internal and external coaxial cylinders are separated by air. The primary radiation is emitted via the slots (each slot is matched to a dipole with the magnetic current source), which are periodically positioned along the antenna’s outer cylinder as depicted in Figure 1a. According to Babinet’s principle, the slots are complementary to the dipole antenna. The far field of the linear dipole [31], is found using:

$$E_{\theta }=\left(j60I_{m}cos\;cos\;\left(klcos\theta \right)\;e^{-jkr}\right)/rsin\theta$$

In the equation, *θ* is the angle between the line direction and the dipole. This means the pattern function of the dipole is the same as the slot antenna.

$$F_{\theta }=\left(cos\;cos\;\left(klcos\theta \right)\;-coskl\right)/sin\theta$$

For idea half wavelength slot, 2l = λ/2, and

$$F_{\theta }=Cos\left(2\pi cos\theta \right)/sin\theta$$

The pattern of the slot antenna is the same as the dipole with the same length, but their elevation plane (E-plane) and omnidirectional plane (H-plane) are exchanged according to the duality principle. Each slot aperture produces horizontally polarized radiation. Four apertures around the circumference complete the antenna, as illustrated in Figure 1a,b, and radiate in an omnidirectional pattern. The SMA connector smoothly converts the TEM modes from SMA to a large antenna assembly with a matching structure that is an optimized inner pin height, as given in Figure 1b.

The diameter of the outer cylinder, the diameter of the feed pin that connects the inner cylinder to the outer part of the antenna, and the length of the slots are what primarily impact the performance of the antenna. The optimal specifications are listed in Table 1.

At UAV GCSs, there are relaxed limitations with regard to size and weight compared to aerial platforms [32]. For military operations, the UAV operator at the GCS is located in a harmless, secured place while the desired information or strategic data from the battlefield is gathered remotely. For such applications, antennas must be capable of withstanding all terrain operational area requirements and should be able to function correctly under extreme weather scenarios. So the required antenna should be mechanically robust and sturdy without external supports as these supports would increase the antenna’s size [4] and result in more drag, which might weaken the antenna’s structure due to rigorous terrain and weather conditions [4,33]. Thus, it is crucial to use a compact, aerodynamic design. There is often a chance that an instance of peak instantaneous power (PIP) happens inside the printed circuit board- (PCB) based feed network. Such an event would easily damage the PCB [34], so the feed must be capable of bearing sudden PIP. The antenna would also be the primary source to channel electrostatic discharge (ESD) and lightning into the electronic systems. An ESD incident would place the functionality and safety of these systems at risk, while a lightning bolt would annihilate them. Keeping the antenna DC grounded is the most feasible and efficient strategy used in combat [35]. This antenna design would circumvent all the problems described above. The axis-symmetrical, all-metal rugged antenna is primarily constructed of brass and is DC-grounded. The solid metal feed network is enclosed inside the antenna’s conformal and compact shape.

## 3. Feed Mechanism

**Figure 2:**
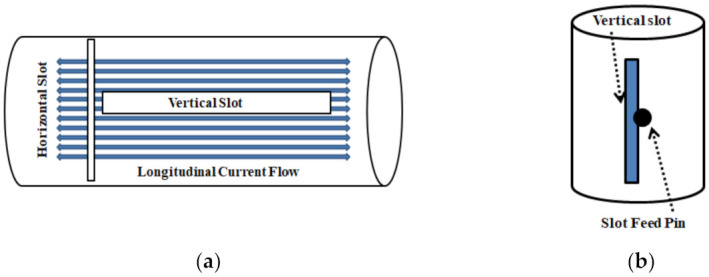
Slot configurations: (**a**) vertical and horizontal slot, (**b**) vertical slot feed.

Horizontal slots induce vertical polarization as they can quickly interrupt the longitudinal surface current on the antenna’s outer surface [36], as seen in Figure 2. Conversely, the longitudinal slots in the antenna’s outer surface cannot be stimulated due to their orientations that are in line with the surface current, and even a short circuit would not modify the flow of the surface current [28]. So it is not easy to produce horizontal polarization using a slot configuration on a coaxial cylinder. In our design, feed pins are inserted to excite the vertical slots, which connect the outer conductor of the oversized coaxial cable with its inner conductor, as shown in Figure 1b. Thus, these slot apertures are energized sideways while the opposing sides are kept floating. The slot is regarded as a dipole having a magnetic current source [29], so the slot is λg/2 long. Normally, the external feed has a built-in problem where it radiates along with the main radiating elements and causes a significant gain ripple in the omnidirectional pattern. Here, we have designed an internal feed that runs inside the radiating part and does not interfere or radiate. As for the actual feeding of the antenna, a standard SMA connector is used for feeding. The SMA connector is a coaxial structure and the antenna designed in this section is also based on coaxial structure, so the matching structure is designed and inserted between the radiation part and the feed part according to impedance transformation of coaxial transmission line [37],

$$Z_{oversize}=Z_{match}(Z_{sma}+jZ_{match}tan\beta T)/\left(Z_{match}+jZ_{sma}tan\beta T\right)$$

where the *T* is the length of the matching pin and *Z_sma_*, *Z_match_*, and *Z_oversize_* are the characteristic impedances of the SMA connector, matching pin, and oversize antenna assembly, respectively. The SMA connector’s inner pin’s optimized height ensures a seamless transition from normal Coaxial TEM mode to oversized TEM cable mode. The slot excitation of the proposed antenna is simple and easy without involving baluns or impedance transformers. Four pins join the inner cylinder to the vertical slots in the antenna’s outer cylinder. The feed and antenna can be conveniently integrated by arranging the manufactured parts together around the central axis.

## 4. Simulation Verification

**Figure 3:**
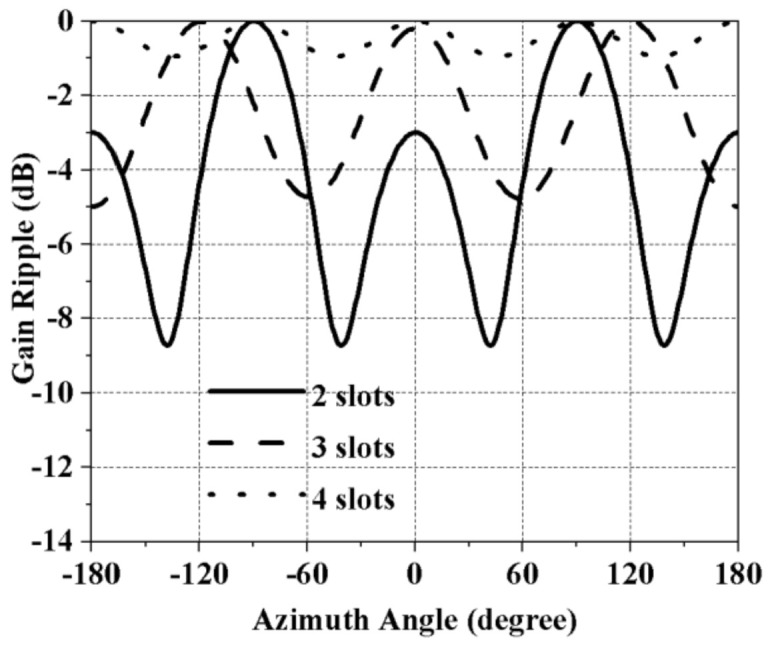
Azimuthal gain vs. the number of slots along the antenna’s circumferential axis.

CST Microwave Studio was been used to simulate and optimize the antenna design. Figure 3 demonstrates the mutual connection between the antenna azimuth gain and the total number of slots along the antenna’s circumference. Each slot radiates a directed pattern. With each increment in the number of slots along the antenna’s circumferential axis, these directional radiations widened, as shown in Figure 3. Four slots made the radiation patterns combine and generate a low-ripple horizontal polarized omnidirectional radiation pattern.

**A. Determination of Pin Diameter and Slot Size Effect**

**Figure 4:**
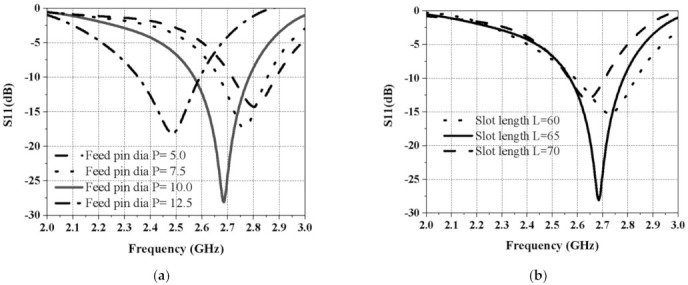
Effect on S_11_ (**a**) by changing the feed pin diameter and (**b**) by changing the slot length.

Figure 4 helps us to see the impact of the slot-feed pin diameter on the antenna input reflection. The change of diameter changed the antenna’s matching, as shown in Figure 4a. Figure 4b depicts how the slot-length variations shifted the antenna resonance region.

**B. Field Verification**

**Figure 5:**
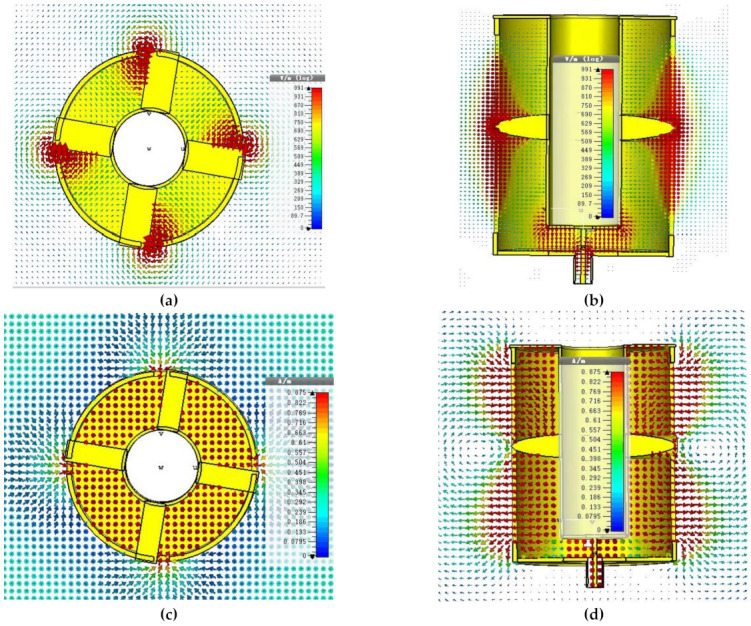
The cross-sectional views of the fields at the SMA connector: (**a**) the electric field, (**b**) the magnetic field cross-sectional views of the fields at the slot-feeding pins, (**c**) the electric field (**d**) the magnetic field.

The field simulations were performed with the help of CST Microwave Studio software. The electric and magnetic fields’ cross-sectional views through the SMA connector are shown Figure 5a,c. The cross-sectional views of the electric and magnetic fields through the pins that connect the inner coaxial cylinder to the slot apertures in the outer coaxial cylinder are shown in Figure 5b,d. At the input SMA connector of the antenna, the electric field is spread radially outward (TEM mode). At the edge of the oversized coaxial antenna assembly near the SMA connector, the electric field is again radially outward as that of the TEM mode. This demonstrates that the SMA connector’s inner pin’s adjusted length effectively converted the connector TEM mode into the oversized coaxial assembly TEM mode, as shown in Figure 5a. As this mode travels toward the pin connected to the slot, the field circulates the slot area. All four slots have the same circulation pattern, which indicates that they are all in phase, as seen in Figure 5b. The electric field steadily travels from the slot middle toward the slot end and thereby emits a horizontally polarized field, as seen in Figure 5a,b. The directional radiation slot patterns are large enough to converge to generate an omnidirectional, horizontally polarized outward wave. Correspondingly, the magnetic fields form closed loops (TEM mode) at the SMA feed and eventually transform to perpendicular loops corresponding to the E field outside the antenna, as clearly visible in the simulated field trajectories in Figure 5b,d.

## 5. Antenna Fabrication and Measurement Result

**Figure 6:**
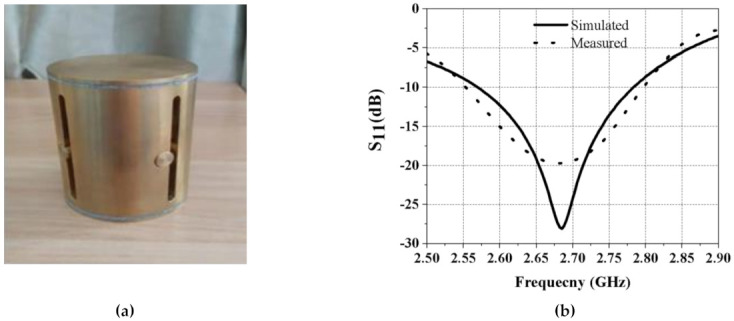
(**a**) The fabricated prototype antenna. (**b**) Simulated and measured S_11_ of the antenna.

An antenna was manufactured using brass for the design validation. This antenna can be assembled using CNC-machined parts or expensive 3D printing. This antenna was built utilizing the first approach. The antenna had a reduced footprint and conformal shape to maintain low air resistance. The simulated and measured antenna test results are discussed in this section. Figure 6a displays an image of the prototype antenna. The input scattering parameter S_11_ of the manufactured antenna was measured with Agilent N5242A VNA’s help. In Figure 6b, the simulated and measured S_11_ are plotted. Measurements were less than −10 dB from 2.5 GHz to 2.8 GHz, which were in good harmony with simulations. The antenna was a reasonably broadband structure (achieved bandwidth of 11.3%).

**Figure 7:**
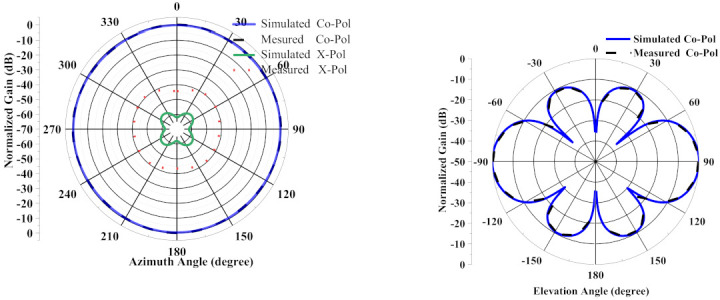

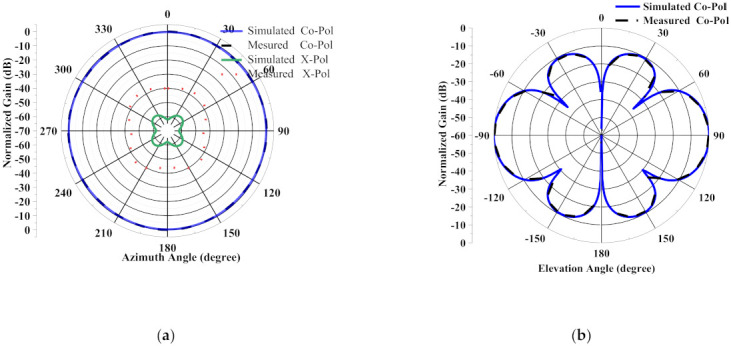
(**a**) Measured and simulated normalized co-polarization and cross-polarization in the omnidirectional plane, or H-plane; top 2.6 GHz; bottom 2.7 GHz. (**b**) Measured and simulated normalized co-polarization in the elevation plane, or E-plane; top 2.6 GHz; bottom 2.7 GHz.

**Figure 8:**
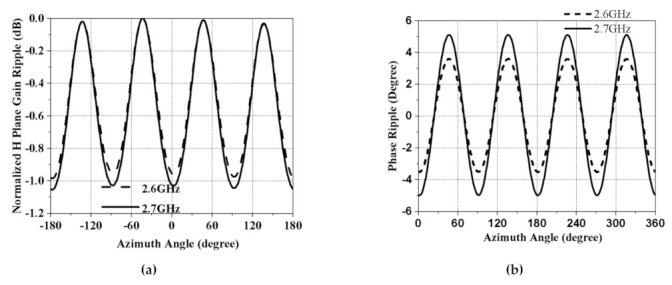
(**a**) Measured gain ripple vs. azimuth angle, (**b**) measured phase angle ripple vs. azimuth angle, at 2.6 GHz and 2.7 GHz, respectively.

Measured and simulated vertical elevation planes and horizontal azimuth planes of the antenna at 2.6 GHz and 2.7 GHz are plotted in Figure 7. Measurements were done in the the compact antenna test range (ATR) of March Microwave Systems B.V., which uses a source antenna that radiates a spherical wavefront and two secondary reflectors to collimate the radiated spherical wavefront into a planar wavefront within the desired test zone where the test antenna is placed and precalibrated standard gain antennas are used to determine the absolute gain of the AUT(antenna under test). The simulated and measured co-polarization (normalized) and cross-polarization (normalized) radiation patterns in the omnidirectional plane (H-plane) are shown in Figure 7a. The 360° radiation at the horizontal plane helps to maintain complete yaw plane operation. The measured cross-polarization levels in the azimuth plane are more than −40 dB down, which agrees with the simulation. Figure 7b depicts the simulated and measured co-polarization (normalized) radiation patterns in vertical elevation (E-plane). Figure 8a,b shows a measured azimuth gain ripple of ±0.5 dB, whereas the azimuth pattern phase ripple is only 10° peak-to-peak. These were measured at 2.6 GHz and 2.7 GHz, respectively. Both affirm the excellent stability of the antenna pattern.

**Figure 9:**
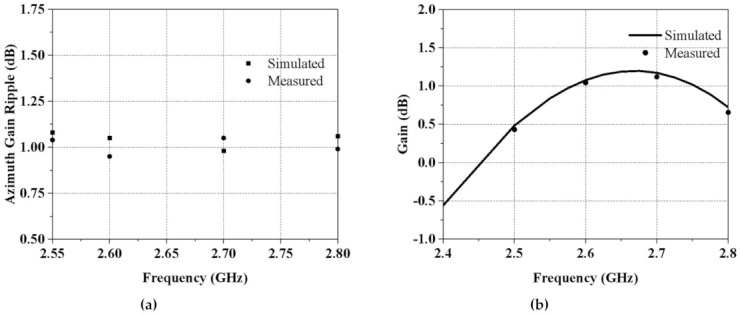
(**a**) Measured and simulated azimuth gain ripple vs. frequency. (**b**) Measured and simulated antenna gain vs. frequency.

In Figure 9a, the measured and simulated azimuth gain ripple are plotted for the entire frequency range for the clear visibility of the gain fluctuations. The maximum peak-to-peak value is 1 dB in the azimuth plane, confirming a good omnidirectionality. Figure 9b illustrates the simulated and measured gain of our antenna. This DC-grounded antenna demonstrated reliable gain within the entire band. These results indicate promising and effective radiation characteristics in the yaw plane, making this antenna an appealing option for GCS applications.

## 6. Comparison

Table 2 compares this study to the prior works published in the literature that also featured horizontal polarization. All works tabulated were designed with external open feed and lack lighting protection capability. The polarization purity was also not so high. This work proposes for the first time an antenna that could produce horizontal polarization utilizing slot radiators and form a stable gain at the azimuth plane like traditional omnidirectional design topologies such as loop or printed dipole antenna arranged in the form of a circle. Due to vertical slot radiations, its cross-polarization levels are extremely low, as shown in the Table. The proposed antenna is novel because it has an internal axis-symmetric feeding system. Due to its enclosed nature, the feed does not radiate and interfere with the main radiating slots. So low-gain ripples in the azimuth plane are achieved as compared to the listed works. Moreover, it is DC-grounded, which is essential for any practical deployment.

## 7. Conclusions

A novel horizontally polarized omnidirectional antenna that is built on the slot structures is introduced in this work. By positioning four vertical slots along the antenna circumference and energizing them with a robust central feeding mechanism, steady gain with improved polarization stability is realized in the antenna’s azimuth axis. The internal axis-symmetric feed network itself radiates no power, hence it does not interfere with the radiating structure. Low ripples appear among the antenna azimuth gain. Steady yaw plane gain with low gain fluctuations enhances coverage area or increases the link efficiency. So it is desirable in numerous ground station-based applications, such as UAV communication and direction finding, to have a low azimuth gain ripple antenna. This antenna also possesses requisite mechanical features, which are crucial for its smooth operation. It has a sturdy, DC-grounded construction that does not need any exterior framework or support. Its conformal and aerodynamic shape minimizes air drag and any corresponding degradation attributed to military operations’ environmental and terrain conditions. Altogether, this may make this antenna a favorable candidate for ground stations.

## Figures and Tables

**Table 1:** Optimal parametric values of the antenna.

Parameter	Value (mm)
Outer Cylinder Diameter A	60
Inner Cylinder Diameter B	25
Slot feed Pin Diameter P	10
Antenna Height H	75
Slot Length L	65
Slot Width W	5
SMA Pin Height T	7.25

**Table 2:** Performance comparison of the proposed antenna with the existing literature.

Reference	Polarization Purity	Gain Ripple (dB)	DC Ground	Feed Type
[17]	11	±5.5	No	Exposed
[18]	20	NA	No	Exposed
[19]	20	1.3	No	Exposed
[20]	25	1.0	No	Exposed
[21]	18	1.5	No	Exposed
[22]	20	1.5	No	Exposed
[23]	15	1.5	No	Exposed
[24]	15	NA	No	Exposed
[25]	20	2.0	No	Exposed
[26]	20	2.1	No	Exposed
Proposed work	40	±0.5	Yes	Enclosed

## Data Availability

Not applicable.
